# Precision dissection strategy for myocutaneous flap in frontotemporal cranioplasty based on tissue biophysical properties

**DOI:** 10.3389/fsurg.2026.1794362

**Published:** 2026-06-05

**Authors:** Li PingGen, X. Zheng, Z. Y. Li, H. Wu, N. F. Wang, Z. G. Leng, L. Huang, W. J. Wu, W. X. Liu, B. H. Li, G. B. Huang

**Affiliations:** Department of Neurosurgery, Yichun People’s Hospital, Yichun, Jiangxi, China

**Keywords:** complications, cranioplasty, myocutaneous flap, precision dissection, surgical strategy, tissue biophysics

## Abstract

**Background:**

Safely and efficiently separating myocutaneous flaps that are densely adherent to the dura mater remains a critical challenge in cranioplasty. Traditional dissection methods and prophylactic implantation of anti-adhesion materials both have limitations. This study aims to propose and validate a “Tissue Biophysics-Based Myocutaneous Flap Dissection Strategy.” By systematically analyzing the biophysical properties of different tissue interfaces and dynamically matching the optimal dissection instruments and techniques, we seek to optimize surgical outcomes.

**Methods:**

We retrospectively analyzed the clinical data of 124 patients who underwent frontotemporal cranioplasty at our institution between January 2020 and September 2025. Patients were divided into a new strategy group (*n* = 55) and a conventional group (*n* = 69). The new strategy group employed blunt mechanical expansion techniques based on the loose subgaleal plane and low-power selective electrothermal dissection techniques based on the impedance difference between the temporalis muscle and the dura mater. The conventional group received standard dissection methods. We compared operative time, intraoperative blood loss, dural tear rate, postoperative drainage, and complication rates between the two groups.

**Results:**

The new strategy group demonstrated significantly lower intraoperative blood loss (128.18 ± 45.32 mL vs. 276.81 ± 68.54 mL, *P* < 0.001), shorter operative time (110.55 ± 18.27 min vs. 162.32 ± 22.65 min, *P* < 0.001), and a reduced dural tear rate (12.7% vs. 39.1%, *P* = 0.002) compared to the conventional group. The incidence of postoperative epidural hematoma was also significantly lower in the new strategy group (10.9% vs. 30.4%, *P* < 0.001). No statistically significant differences were found in the rates of other complications such as infection or epilepsy.

**Conclusion:**

The proposed “Tissue Biophysics-Based Myocutaneous Flap Dissection Strategy” integrates tissue biophysical principles (e.g., interfacial compliance, electrical impedance differences) into surgical decision-making, aiming to provide a practical surgical strategy. This surgical strategy appears to enhance the safety and efficiency of cranioplasty while reducing the risk of key complications in this patient cohort.

## Introduction

Cranioplasty is a crucial neurosurgical reconstructive procedure for repairing skull defects following decompressive craniectomy, aiming to restore intracranial homeostasis and craniofacial contour (1–3). However, postoperative scar tissue formation often leads to dense adhesions between the myocutaneous flap and the dura mater. This poses significant challenges during secondary dissection, including difficult-to-control intraoperative hemorrhage, prolonged operative time, and a high incidence of incidental dural tears, all of which severely compromise surgical safety and patient outcomes (4–6).

In current clinical practice, the separation of the myocutaneous flap relies heavily on the surgeon's individual experience, utilizing scalpels, surgical scissors, or electrocautery. These methods are essentially forms of “blind dissection” that universally neglect the fundamental histological, mechanical, and electrophysiological differences between the loose connective tissue plane beneath the galea aponeurotica and the fibrous interface between the temporalis muscle and the dura mater. Consequently, they frequently result in loss of the correct dissection plane, significant tissue trauma, and persistently high rates of dural violation, which represent a primary cause of perioperative complications. Although the prophylactic use of anti-adhesion materials can partially mitigate the adhesion problem (7, 8), it carries potential risks such as infection and foreign body reactions (9–12).

Therefore, addressing this clinical challenge requires more than merely “meticulous anatomical dissection.” There is an urgent need to establish a systematic strategy capable of dynamically and precisely matching dissection instruments and techniques based on the intraoperative biophysical properties of the tissues. This necessitates a surgical strategy that integrates a tissue biophysics perspective with standardized procedures. Accordingly, this study proposes a “Precision Dissection Strategy for Myocutaneous Flaps Based on Tissue Biophysical Properties.” Its core premise is to transform flap separation from a skill-dependent maneuver into a standardized, decision-driven, and instrument-adapted workflow guided by interfacial property analysis. Tailored to the distinct characteristics of the galea-dura interface (featuring a natural loose plane) and the temporalis muscle-dura interface (lacking a natural plane, with heterogeneous tissue impedance), the strategy respectively employs “blunt mechanical expansion” and “low-power selective electrothermal dissection” techniques. Through a retrospective cohort analysis, this study aims to validate the comprehensive advantages of this strategy in enhancing surgical efficiency, ensuring operative safety, and preserving critical anatomical structures. It seeks to provide a scientific, safe, and standardizable approach for myocutaneous flap dissection in cranioplasty.

## Methods

### Ethics approval and consent to participate

This study was performed in the Department of Neurosurgery, Yichun People's Hospital, and the study protocol was approved by the hospital's Ethics Committee. A retrospective review was conducted in patients who underwent frontotemporal cranioplasty from January 2020 to September 2025. Inclusion criteria: 1) Unilateral frontotemporal decompressive craniectomy due to traumatic brain injury, spontaneous intracerebral hemorrhage, or large-area cerebral infarction, followed by cranioplasty; 2) Absorbable dural substitute materials were used during the decompressive craniectomy; 3) Age 18–75 years; 4) Complete clinical data. Exclusion criteria: 1) Non-frontotemporal skull defect location; 2) Cranioplasty performed with temporalis muscle exclusion technique; 3) Preoperative use of antiplatelet/anticoagulant agents or presence of coagulation disorders; 4) Active infection, poor wound healing, or intracranial infection in the surgical area. Baseline patient demographics, clinical characteristics, surgical sarameters and complication rates are shown in Tables 1–3. All the patients or their family members provided informed consent, which contained a detailed description of the purpose of this study. All the information of the cases was handled and made anonymous according to ethical and legal standards.

**Table 1:** Patient demographics and clinical characteristics.

Characteristic	New Strategy Group (*n* = 55)	Conventional Group (*n* = 69)	Statistic	*P*-value
Gender (Male, n)	44 (80.0%)	48 (69.6%)	*χ*^2^ = 1.238	0.266
Age (years)	52.0 ± 11.0	50.6 ± 12.3	t = 0.692	0.491
Timing of Surgery (days)	78.0 [70.0–95.5]	89.0 [76.0–100.0]	Z = −1.687	0.093
Implant Material (n)			χ^2^ = 1.215	0.272
- PEEK	30 (54.5%)	29 (42.0%)		
- Titanium Mesh	25 (45.5%)	40 (58.0%)		

**Table 2:** Comparison of surgical parameters between two groups.

Metric	New Strategy Group (*n* = 55)	Conventional Group (*n* = 69)	Statistic	*P*-value
Intraoperative Blood Loss (mL)	128.18 ± 45.32	276.81 ± 68.54	t = −14.236	<0.001
Operation Time (minutes)	110.55 ± 18.27	162.32 ± 22.65	t = −14.892	<0.001
Dural Breach (n)	7 (12.7%)	27 (39.1%)	χ^2^ = 9.563	0.002
Postop 48-h Drainage (mL)	238.18 ± 32.65	312.46 ± 45.82	t = −10.985	<0.001

**Table 3:** Comparison of postoperative complication rates .

Complication	New Strategy Group (*n* = 55)	Conventional Group (*n* = 69)	Test	Statistic	*P*-value
Epidural Hematoma (n)	6 (10.9%)	21 (30.4%)	Pearson χ^2^	χ^2^ = 5.618	0.017
Epidural Effusion (n)	16 (29.1%)	30 (43.5%)	Pearson χ^2^	χ^2^ = 2.134	0.144
Infection (n)	1 (1.8%)	0 (0.0%)	Fisher's Exact	-	0.444
Epilepsy (n)	1 (1.8%)	2 (2.9%)	Fisher's Exact	-	1.000
re-operation	0 (0.0%)	1 (1.5%)	Fisher's Exact	-	1.000

### Patient grouping and surgical team

The conventional group consisted of patients treated between January 2020 and December 2023, during which traditional dissection methods were used. The new strategy group consisted of patients treated between January 2024 and September 2025, after the new dissection strategy was introduced and standardized in our department. The first 10 cases of the new strategy were excluded from analysis to minimize the impact of the learning curve; only the subsequent 55 cases were included in the new strategy group. All procedures in both groups were performed by the same team of three senior neurosurgeons, each with more than 10 years of experience in cranioplasty.

### Precision dissection strategy for myocutaneous flap in frontotemporal cranioplasty based on tissue biophysical properties

**Figure 1:**
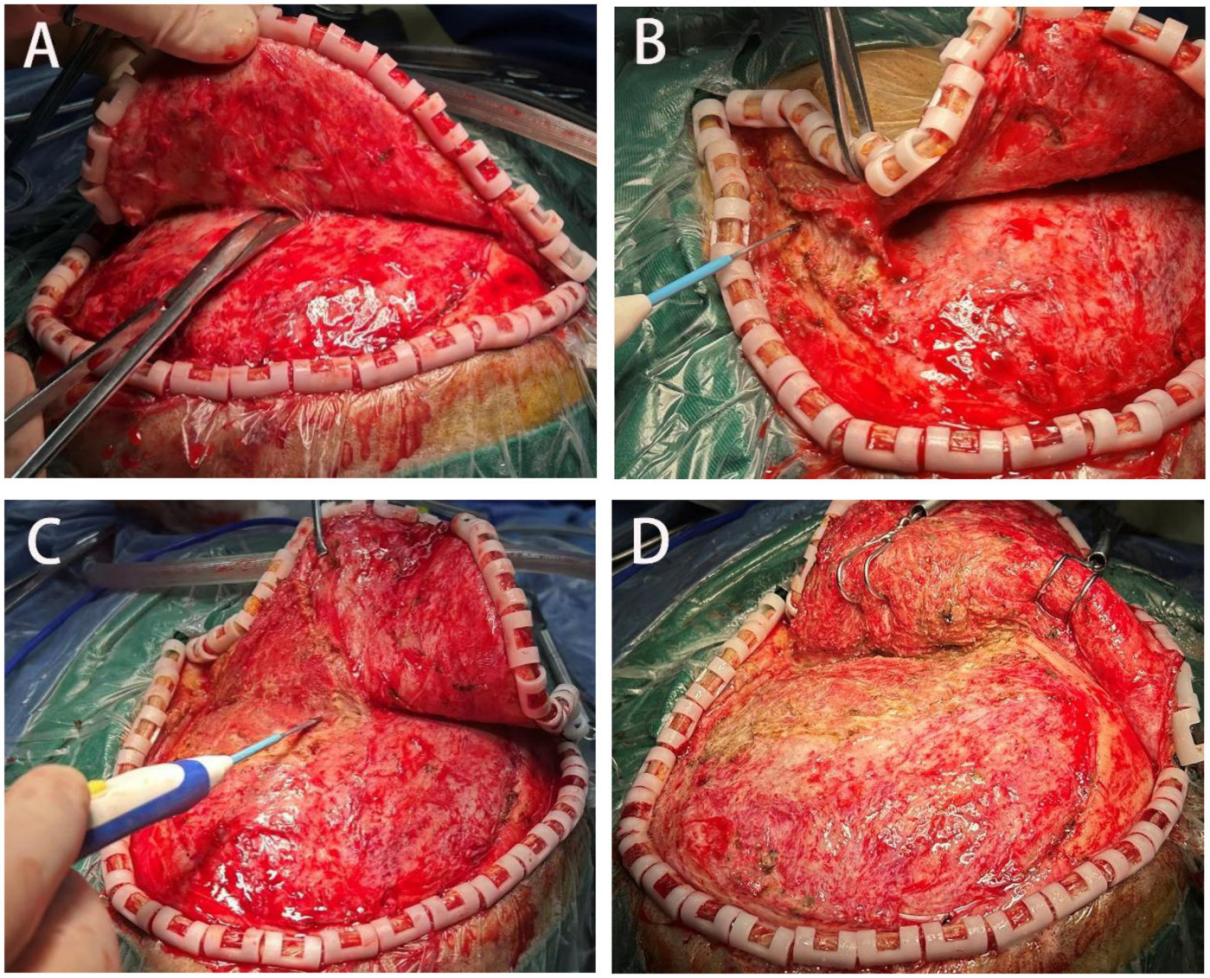
**(A)**: blunt dissection with tissue scissors in the subgaleal plane of the frontoparietal region. **(B)**: Fine dissection with low-power monopolar electrocautery at the temporalis-dura interface in the temporal region. C: Dissection proceeding superiorly to the temporal line and inferiorly. D: Completed flap elevation.

1. Frontoparietal Galea-Dura Interface Dissection: At the frontal bone edge, the subgaleal plane was identified. Blunt dissection was performed using tissue scissors close to the galea layer, lifting the dura and its surface scar tissue *en bloc*, proceeding until the temporal line (convergence of galea, dura, and temporalis fascia) (Figure 1A).
2. Temporal Temporalis-Dura Interface Dissection: At the temporal bone edge, the temporalis-dura interface was identified. Fine dissection was performed using a monopolar electrocautery at a low power setting (15 W) (Figure 1B). The electrode tip was kept nearly parallel to the dura surface to meticulously strip muscle fibers adherent to the dura, proceeding superiorly to meet the frontoparietal flap at the temporal line and inferiorly to the lower bone edge (Figures 1C,D). It is worth noting that this low-power electrocautery technique is applied only at the temporalis-dura interface, where the impedance difference between muscle and dura facilitates selective dissection. Conversely, the galeal flap is elevated using blunt scissors to achieve safer, more efficient dissection.

Technical Specifications: Monopolar electrosurgery was performed using an electrosurgical unit manufactured by Changzhou Yanling Electronic Equipment Co., Ltd. (Changzhou, Jiangsu, China) with a standard blade electrode. The device was set to ‘Coagulation’ mode at 15 Watts. A standard adhesive patient return electrode (grounding pad) was placed on the anterior thigh. The electrode tip was moved in a gentle ‘painting’ or ‘gliding’ motion parallel to the dural surface to limit thermal spread.

### Definitions

Dural breach was defined as any intraoperative disruption of dural integrity, including both overt large tears and minor dural ruptures without obvious cerebrospinal fluid leakage. All identified dural breaches were repaired with suture and recorded. This comprehensive definition was applied uniformly to both groups to ensure consistency and objectivity in outcome assessment.

### Statistical method

SPSS 22.0 was used for analysis. Normally distributed continuous variables are presented as mean ± standard deviation (X ± s) and compared using independent samples t-test. Non-normally distributed variables are presented as median (interquartile range) and compared using Mann–Whitney U test. Categorical data are presented as n (%) and compared using Chi-square test or Fisher's exact test. *P* < 0.05 was considered statistically significant. For continuous variables, effect sizes (Cohen's d) and 95% confidence intervals (CIs) for mean differences were calculated. For binary outcomes, multivariable logistic regression was performed to identify independent risk factors, with results presented as adjusted odds ratios (ORs) and 95% CIs. Variables included in the regression model were age, gender, implant material, timing of surgery (log-transformed), and surgical strategy (new vs. conventional).

## Results

### Patient demographics and clinical characteristics

A total of 124 patients were included (new strategy group: *n* = 55; conventional group: *n* = 69). No statistically significant differences were found between the two groups regarding gender, age, timing of surgery, or implant material (PEEK vs. titanium mesh) (all *P* > 0.05), indicating comparability (Table 1).

### Comparison of surgical parameters between two groups

The new strategy group demonstrated significantly lower intraoperative blood loss (mean difference: −148.63 mL, 95% CI: −171.20 to −126.06, Cohen’s d = 2.48, *P* < 0.001), shorter operative time (mean difference: −51.77 min, 95% CI: −59.30 to −44.24, Cohen’s d = 2.51, *P* < 0.001), and reduced postoperative 48-hour drainage (mean difference: −74.28 mL, 95% CI: −88.01 to −60.55, Cohen’s d = 1.91, *P* < 0.001) compared with the conventional group (Table 2).

### Comparison of postoperative complication rates

The incidence of postoperative epidural hematoma was significantly lower in the new strategy group (*P* = 0.017). No statistically significant differences were found between the two groups regarding the incidence of epidural effusion, infection, or epilepsy. Among patients with postoperative epidural hematoma, surgical evacuation was required in 1 of 21 patients (4.8%) in the conventional group, while none of the 6 patients (0%) in the new strategy group required re-operation(all *P* > 0.05, Table 3).

Multivariable logistic regression, adjusting for age, gender, timing of surgery, and implant material, showed that the new strategy was independently associated with a reduced risk of dural breach (adjusted OR = 0.23, 95% CI: 0.09–0.61, *P* = 0.003) and postoperative epidural hematoma (adjusted OR = 0.28, 95% CI: 0.10–0.79, *P* = 0.016).

### Illustrative case of new strategy

**Figure 2:**
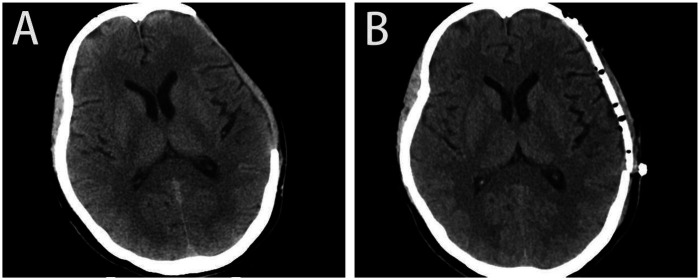
**(A)**: preoperative CT showing left frontotemporal skull defect. **(B)**: Postoperative CT showing well-positioned PEEK implant without intracranial hemorrhage/effusion.

A 56-year-old female underwent cranioplasty 2 months post-craniectomy for TBI. Preoperative CT showed left frontotemporal skull defect (Figure 2A). The zoning-based layered dissection technique was applied. Intraoperative blood loss was 100 mL, operative time 87 min. Postoperative CT showed no intracranial hemorrhage/effusion (Figure 2B). At 1-month follow-up, GOS was 5 with well-healed incision.

## Discussion

This retrospective analysis of 124 patients undergoing frontotemporal cranioplasty suggests that the “Precision Dissection Strategy for Myocutaneous Flaps Based on Tissue Biophysical Properties” systematically and is associated with improved perioperative outcomes compared to traditional dissection methods. This achievement represents more than a technical refinement; it embodies a novel diagnostic and therapeutic paradigm that integrates tissue biophysical principles into surgical decision-making. Its efficacy is rooted in the scientific recognition and strategic exploitation of the inherent differences in properties between tissue interfaces, achieving a three-dimensional optimization encompassing anatomical safety, operative efficiency, and tissue preservation.

### Biomechanistic rationale for strategy efficacy and data corroboration

The present strategy differs from conventional macro-anatomical approaches by emphasizing tissue biophysical properties. The observed therapeutic advantages can be interpreted in light of established biophysical principles, providing a theoretical rationale that is supported by our data.

At the subgaleal interface**,** the strategy relies on the mechanical properties of this plane—characterized by low shear resistance and high compliance as a natural loose connective tissue space (13, 14). The use of closed scissors for precise blunt dissection is, in essence, a controlled mechanical expansion process. This maneuver theoretically respects the inherent biomechanical properties of the plane, allowing for the *en bloc* and atraumatic separation of the tough galea aponeurotica from the adherent underlying dura and scar tissue, potentially minimizing sharp transection injury to the intervening microvascular network. The precise application of this biophysical principle is directly reflected in our core finding: intraoperative blood loss in the new-strategy group was significantly reduced to 128.18 ± 45.32 mL, representing a 53.7% decrease compared to the conventional group (276.81 ± 68.54 mL, *P* < 0.001).

At the temporalis muscle-dura interface**,** which lacks a natural cleavage plane and features vascular, fibromuscular adhesions, the theoretical basis lies in the precise application of the principle of differential tissue electrical impedance (15, 16). Skeletal muscle cells, rich in electrolytes, exhibit low electrical impedance, whereas the dura mater, composed primarily of dense collagen fibers, exhibits high impedance. The low-power (15W) monopolar electrocautery fine-dissection technique employed in this study is conceptually based on this differential impedance: it is postulated that at the set power, current preferentially flows through and concentrates in the low-impedance muscle tissue, generating localized Joule heat sufficient for protein denaturation and cellular disruption (achieving selective cutting with simultaneous coagulation), while causing minimal damage to the high-impedance collagen structure of the dura. This effect may be further refined by a “gliding” technique, where the electrode tip is moved parallel to the dural surface, theoretically confining the thermal effect to the most superficial layer of muscular attachment and enabling a effective selective thermodissection. Consistent with this theoretical rationale, the incidence of incidental dural tears was reduced from 39.1% in the conventional group to 12.7% (*P* = 0.002), and the rate of postoperative symptomatic epidural hematoma also decreased from 30.4% to 10.9% (*P* < 0.001). This approach enables the surgeon to use electrocautery more selectively based on differential tissue impedance, but the quality of dissection ultimately depends on the surgeon's skill and experience.

### Core concepts of the surgical strategy

The principal contribution of this study lies in the construction and preliminary validation of an integrative surgical strategy, the multifaceted clinical value of which is comprehensively supported by our data:

Conceptualbasis: Proposing a new philosophy of “tissue property-guided” precision surgery, shifting the paradigm of flap dissection from experience-dependent maneuvers to principle-driven procedures. It should be noted that the individual technical maneuvers employed in this strategy—blunt dissection in the subgaleal plane and low-power electrocautery at the temporalis–dura interface—are established practices in neurosurgery (17, 18). The primary contribution of this study lies not in the invention of novel tissue effects, but in their systematic integration into a coherent new surgical strategy guided by tissue property analysis. The study data show that this reproducible, standardized framework significantly reduced the mean operative time (110.55 ± 18.27 min vs. 162.32 ± 22.65 min, *P* < 0.001), demonstrating its pronounced advantage in enhancing surgical efficiency and facilitating standardized dissemination.
Methodologicalfeatures: Establishing a standardized decision-making workflow of “interface identification → property analysis → instrument matching.” This framework not only explains the mechanisms behind the significant reductions in intraoperative bleeding and dural injury from first principles but, through its structured procedural flow, also reduces reliance on individual surgeon experience, markedly improving the teachability and clinical reproducibility of the technique.
Clinical relevance: Achieving the synergistic optimization of perioperative safety and long-term functional preservation. Beyond the significant improvement in immediate safety metrics, this strategy provides a critical technical foundation for preventing postoperative temporalis muscle atrophy and temporal hollowing by precisely preserving the neurovascular supply to the muscle (19–23). This addresses the high-level clinical demand of modern neurosurgery for “equal emphasis on functional and aesthetic outcomes,” marking a transition from merely pursuing surgical success to concurrently optimizing both short-term safety and long-term functional-aesthetic results.

### Study limitations and future directions

As a single-center retrospective exploratory study, it has certain limitations: First, the non-randomized, sequential grouping introduces potential chronology bias, as the conventional and new strategy groups were treated in different time periods. Although we standardized the technique and excluded the initial learning curve cases, residual bias due to temporal improvements in surgical experience or perioperative care cannot be fully excluded.; second, the degree of preoperative flap-dura adhesion was not systematically classified or quantified in the retrospective records, which precludes subgroup analysis based on adhesion severity; third, the follow-up period is relatively short, precluding a systematic assessment of long-term functional and craniofacial aesthetic outcomes. Fourth, while the surgical strategy is conceptually grounded in biophysical principles (e.g., tissue impedance and compliance), this study provides only clinical outcome data. Direct biophysical measurements—such as real-time tissue impedance monitoring, *ex vivo* thermal mapping, or histological assessment of thermal spread—were not performed. Therefore, the mechanistic discussion regarding selective energy delivery remains a theoretical interpretation of the observed clinical outcomes and should be viewed as a rationale rather than a proven biophysical finding. Future research should aim to obtain high-level evidence through prospective, multicenter randomized controlled trials.

## Conclusion

In summary, this study suggests that applying a tissue biophysics-guided precision dissection strategy in frontotemporal cranioplasty can safely and efficiently address the challenge of dissecting dense adhesions between the frontotemporal myocutaneous flap and the dura mater. By integrating the differential mechanical and electrophysiological properties of distinct tissue interfaces with appropriate dissection instruments and techniques, this strategy provides a practical surgical approach. It significantly reduces key risks—including intraoperative blood loss, operative time, dural violation, and postoperative hematoma—and, by preserving critical vascular and muscular structures, may help improve patients’ long-term functional and aesthetic outcomes. Further clinical experience is warranted to refine this strategy and evaluate its broader applicability.

## Data Availability

The original contributions presented in the study are included in the article/supplementary material, further inquiries can be directed to the corresponding author.
